# Calibration of Binocular Vision Sensors Based on Unknown-Sized Elliptical Stripe Images

**DOI:** 10.3390/s17122873

**Published:** 2017-12-13

**Authors:** Zhen Liu, Suining Wu, Yang Yin, Jinbo Wu

**Affiliations:** 1 Key Laboratory of Precision Opto-mechatronics Technology, Ministry of Education, Beihang University, No. 37 Xueyuan Road, Haidian District, Beijing 100191, China; liuzhen008@buaa.edu.cn (Z.L.); sunnywu1992@buaa.edu.cn (S.W.); sy1417333@buaa.edu.cn (Y.Y.); 2 School of Naval Architecture & Ocean Engineering, Huazhong University of Science & Technology, 1037 Luoyu Road, Wuhan 430074, China; 3 Hubei Key Laboratory of Naval Architecture & Ocean Engineering Hydrodynamics (HUST), Huazhong University of Science & Technology, Wuhan 430074, China

**Keywords:** calibration, binocular vision sensor, unknown-sized elliptical stripe

## Abstract

Most of the existing calibration methods for binocular stereo vision sensor (BSVS) depend on a high-accuracy target with feature points that are difficult and costly to manufacture. In complex light conditions, optical filters are used for BSVS, but they affect imaging quality. Hence, the use of a high-accuracy target with certain-sized feature points for calibration is not feasible under such complex conditions. To solve these problems, a calibration method based on unknown-sized elliptical stripe images is proposed. With known intrinsic parameters, the proposed method adopts the elliptical stripes located on the parallel planes as a medium to calibrate BSVS online. In comparison with the common calibration methods, the proposed method avoids utilizing high-accuracy target with certain-sized feature points. Therefore, the proposed method is not only easy to implement but is a realistic method for the calibration of BSVS with optical filter. Changing the size of elliptical curves projected on the target solves the difficulty of applying the proposed method in different fields of view and distances. Simulative and physical experiments are conducted to validate the efficiency of the proposed method. When the field of view is approximately 400 mm × 300 mm, the proposed method can reach a calibration accuracy of 0.03 mm, which is comparable with that of Zhang’s method.

## 1. Introduction

Calibration of stereo vision sensors is an essential step of vision measurement [1,2,3]. Vision sensors with high calibration accuracy usually guarantee high measurement accuracy. Vision measurement is mainly conducted to complete the 3D reconstruction of the measured objects. According to the measuring principle, the vision measurement system can be divided into three major categories: (1) line-structured light measurement system; (2) binocular stereo vision measurement system; (3) multi-camera stereo vision measurement system. When adopting the line-structured light method, extraction accuracy of the center of the light stripe affects the measurement accuracy [4,5]. Light scattering occurs when the projection angle between the light plane and the object is relatively large. As a result, calibration and measurement accuracy decline. The multi-camera stereo vision measurement system can implement online vision measurement with a large field of view and multi-viewpoints, and it is equivalent to multi-pair binocular stereo vision sensors (BSVSs) [6]. Therefore, research on the calibration of BSVS is of great significance.

To date, research on the calibration of BSVS mainly focused on the different forms of high-accuracy targets, including 1D [7,8], 2D [9], and 3D targets [10,11]. Zhao et al. [12] proposed a method based on a 1D target with two feature points of known distance. Compared with Zhang’s method [13], which is also based on a 1D target, Zhao’s method not only improves the calibration accuracy of intrinsic parameters but also implements the extrinsic parameter calibration of BSVS. Zhang’s method [14] using the planar checkerboard target has made a remarkable impact on the study of camera calibration. Other methods using rectification error optimization [15] and perpendicularity compensation [16] have been proposed to improve calibration accuracy. 

To achieve high-accuracy calibration under complex circumstances, different forms of targets are utilized in the calibration of BSVS. A calibration method based on spot laser and parallel planar target is proposed to improve calibration under complex light conditions [17]. This method does not rely on feature points with known distance or size. In each shot, only one spot is projected on the target, resulting in low efficiency in online measurement. Given that random noise is inevitable, this method cannot guarantee high accuracy due to the location uncertainty of feature points in a picture. Wu et al. [18] proposed a global calibration method based on vanishing features of a target. In addition, the specially designed target is constructed of two mutually orthogonal groups of parallel lines with known lengths. Zhang et al. [19] proposed a novel method based on spherical target images with certain size, which implements synchronous calibration of a multi-camera system. At present, the spherical target with extremely high quality is hard to manufacture. Considering the noise, unideal light conditions and other factors, using a spherical target to calibrate does not guarantee high accuracy [20,21].

From the abovementioned methods, accuracy of the distance of feature points or the size of the target is a common requirement. In addition, accuracy of the requisite sizes greatly affects the calibration accuracy of BSVS. To solve the problem presented above, this study introduces a novel calibration method that does not rely on specific feature points and works efficiently under complex conditions. The proposed method adapts a ring laser to project an elliptical stripe on the parallel planar target. During the calibration, Zhang’s method is primarily utilized to obtain the intrinsic parameter of two cameras. The elliptical stripes are then used as the medium to solve the extrinsic parameters. Finally, the optimal solutions of calibration results are obtained via non-linear optimization.

The remainder of this paper is organized as follows. Section 2 mainly describes the mathematical model of BSVS, the algorithm principles, realization procedure, and other details of the proposed method. Section 3 discusses other expansive forms of the proposed method, as well as its relevant performance under complex lighting conditions. Section 4 presents the simulation and real data experiments conducted to validate the effectiveness of the proposed method. Section 5 states the conclusions of our work.

## 2. Principle and Methods

### 2.1. Mathematical Model of BSVS

**Figure 1:**
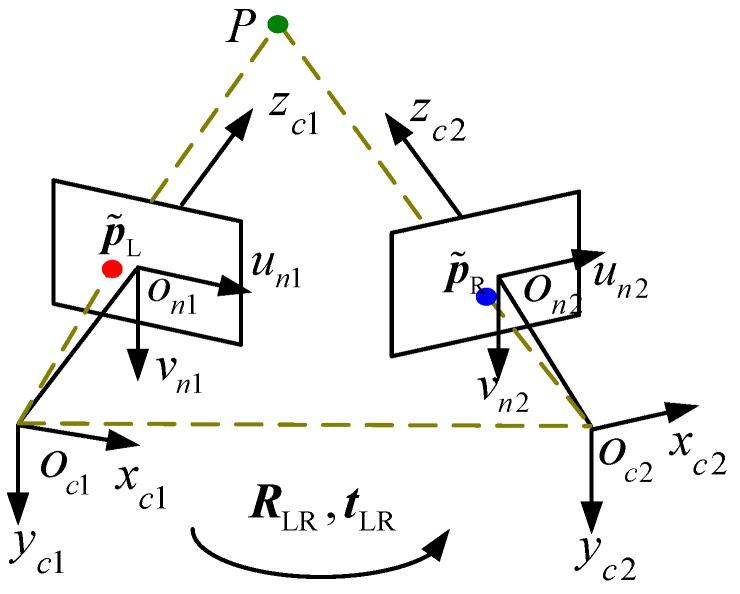
Binocular stereo vision model.

As shown in Figure 1, the coordinate systems of the left and right cameras are $O_{c1}x_{c1}y_{c1}z_{c1}$ and $O_{c2}x_{c2}y_{c2}z_{c2}$, respectively. ${\tilde{p}}_{L}={\left[\begin{array}{ccc} u_{L} & v_{L} & 1 \end{array}\right]}^{T}$ and ${\tilde{p}}_{R}={\left[\begin{array}{ccc} u_{R} & v_{R} & 1 \end{array}\right]}^{T}$ are homogeneous coordinates of non-distorted images of point *P* in the coordinate system of the image by the left and right cameras, respectively. The transformation matrix from the coordinate system of the left camera to that of the right camera is $T_{\mathrm{LR}}=\left[\begin{array}{cc} R_{\mathrm{LR}} & t_{\mathrm{LR}}\\ 0 & 1 \end{array}\right]$, where $R_{\mathrm{LR}},t_{\mathrm{LR}}$ are the rotation matrix and translation vector, respectively. $r_{\mathrm{LR}}$ is the Rodrigues’ representation of the rotation matrix $R_{\mathrm{LR}}$.

The spot *P* is projected by the BSVS. The binocular stereo vision model is used to calculate the 3D coordinates $q_{L}=[\begin{array}{cccc} x_{L} & y_{L} & z_{L} & 1 \end{array}]$ of point *P* in $O_{c1}x_{c1}y_{c1}z_{c1}$:

(1) $$\{\begin{array}{l} \rho_{L}{\tilde{p}}_{L}=K_{L}\left[\begin{array}{cc} I_{3\times 3} & 0_{3\times 1} \end{array}\right]q_{L}\\ \rho_{R}{\tilde{p}}_{R}=K_{R}\left[\begin{array}{cc} R_{\mathrm{LR}} & t_{\mathrm{LR}} \end{array}\right]q_{L} \end{array}$$

where $K_{L}$ and $K_{R}$ are the matrices of intrinsic parameters of the left and right cameras, respectively. $K=\left[\begin{array}{ccc} a_{x} & \gamma & u_{0}\\ 0 & a_{y} & v_{0}\\ 0 & 0 & 1 \end{array}\right]$, where $u_{0}$ and $v_{0}$ are the coordinates of the principal point, $a_{x}$ and $a_{y}$ are the scale factors in the image *u* and *v* axes, and γ is the skew of the two image axes.

### 2.2. Algorithm Principle

**Figure 2:**
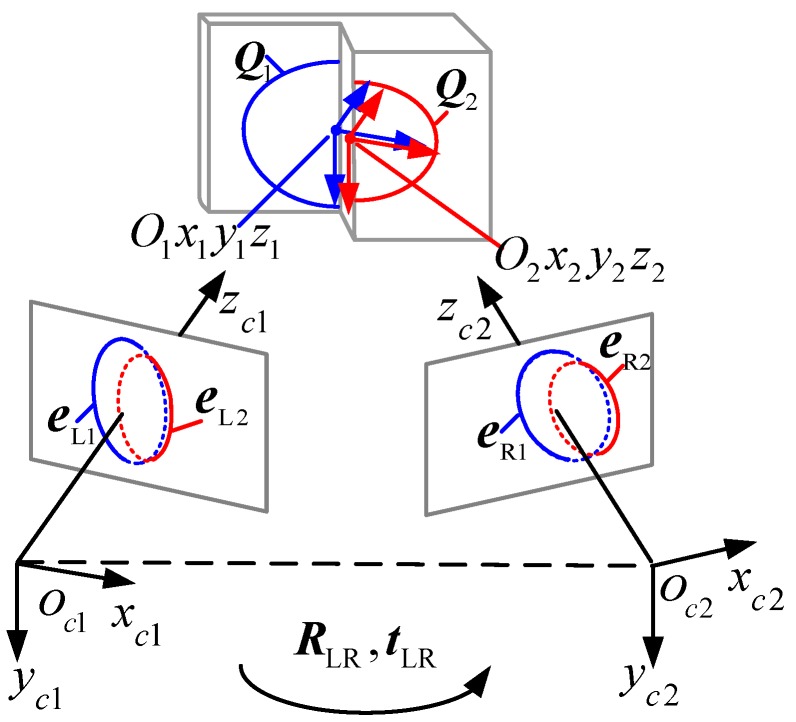
Calibration process of the binocular stereo vision sensor.

The calibration process of the proposed method is shown in Figure 2. In our case, a single ring laser projector and a double parallel planar target are utilized to generate the elliptical stripes as illustrated in Figure 2. In addition, the distance between the two parallel planes is constrained.

As shown in Figure 2, $Q_{j}(j=1,2)$ are the two elliptical stripes projected on the two parallel planes. $Q_{j}=\left[\begin{array}{ccc} 1/\beta_{j}^{2} & 0 & 0\\ 0 & 1/\alpha_{j}^{2} & 0\\ 0 & 0 & -1 \end{array}\right]$ is the expression of the elliptical stripe in space, $2\alpha_{j}$ is the major axis of the *j*-th ellipse, and $2\beta_{j}$ is the minor axis of the *j*-th ellipse. We assume that $O_{j}x_{j}y_{j}z_{j}$ is the coordinate of the *j*-th ellipse in space. For $O_{j}x_{j}y_{j}z_{j}$, the *y*-axis is the major axis of $Q_{j}$ the *x*-axis is the minor axis of $Q_{j}$, and the origin is the center of $Q_{j}$ in space. The projections of $Q_{j}$ in the left and right cameras are denoted as $e_{Lj}$ and $e_{Rj}$, respectively. $R_{Lj}$ and $t_{Lj}$ are the rotation matrix and translation vector from $O_{j}x_{j}y_{j}z_{j}$ to $O_{c1}x_{c1}y_{c1}z_{c1}$, respectively. $R_{Rj}$ and $t_{Rj}$ are the rotation matrix and translation vector from $O_{j}x_{j}y_{j}z_{j}$ to $O_{c2}x_{c2}y_{c2}z_{c2}$, respectively. $R_{\mathrm{LR}}$ and $t_{\mathrm{LR}}$ are the rotation matrix and translation vector from $O_{c1}x_{c1}y_{c1}z_{c1}$ to $O_{c2}x_{c2}y_{c2}z_{c2}$, respectively. All of the coordinate frames generated by the intersection of the parallel plane and conical surface projected by the single ring laser projector are parallel to each other, that is, $R_{L1}=R_{L2}$ and $R_{R1}=R_{R2}$. Notably, the two elliptical stripes captured in each case have the following properties:

1. he ratios of the minor axis to the major axis $k=\beta_{j}/\alpha_{j}$ are equivalent.
2. The minor axis of the major axis of one elliptical stripe is parallel to that of the other elliptical stripe. The angles between the minor axis and the major axis of these two elliptical stripes are equivalent.

#### 2.2.1. Solving $R_{\mathrm{LR}}$

As shown in Equation (2), $e_{Lj}$ and $Q_{j}$ are 3 × 3 matrices. According to multi-view geometry foundation [22], the relationship between $e_{Lj}$ and $Q_{j}$ is as following:

(2) $$\left\{ \begin{array}{l} p^{T}e_{Lj}p=0\\ q^{T}Q_{j}q=0 \end{array}\right.$$

where $p={\left[\begin{array}{ccc} u & v & 1 \end{array}\right]}^{T}$ is the undistorted image homogeneous coordinate of the point on the *j*-th elliptical stripe under $O_{c1}x_{c1}y_{c1}z_{c1}$, and $q={\left[\begin{array}{ccc} x & y & 1 \end{array}\right]}^{T}$ is the coordinate of the point on the *j*-th elliptical stripe under $O_{j}x_{j}y_{j}$.

Combining Equation (2) and the camera model, we have:

(3) $$\rho_{j}Q_{j}={\left(K_{L}[{\begin{array}{ccc} r_{1} & r_{2} & t \end{array}}_{Lj}]\right)}^{T}e_{Lj}K_{L}[{\begin{array}{ccc} r_{1} & r_{2} & t \end{array}}_{Lj}]$$

where $\rho_{j}$ represents the non-zero scale factors, and $r_{j}$ denotes the *j*-th column of the rotation matrix $R_{Lj}$. $K_{L}$ represents the intrinsic parameter of the left camera and is obtained using Zhang’s method.

According to Equation (3), the equation relating $e_{Lj}$ to $Q_{j}$ is obtained in Equation (4):

(4) $$\rho_{j}Q_{j}=\rho_{j}\left(\begin{array}{ccc} 1/\beta_{j}^{2} & 0 & 0\\ 0 & 1/\alpha_{j}^{2} & 0\\ 0 & 0 & -1 \end{array}\right)=\left(\begin{array}{ccc} r_{1}^{T}W_{j}r_{1} & r_{1}^{T}W_{j}r_{2} & r_{1}^{T}W_{j}t_{Lj}\\ r_{2}^{T}W_{j}r_{1} & r_{2}^{T}W_{j}r_{2} & r_{2}^{T}W_{j}t_{Lj}\\ t_{Lj}^{T}W_{j}r_{1} & t_{Lj}^{T}W_{j}r_{2} & t_{Lj}^{T}W_{j}t_{Lj} \end{array}\right)$$

where $W_{j}=K_{L}^{T}e_{Lj}K_{L}$.

For two elliptical stripes located on the target, we have two equations in the form of Equation (4). According to the property of the matrix in Equation (4), equations related to the two elliptical stripes can be decomposed into the following 12 equations:

(5) $$\begin{array}{l} r_{1}^{T}W_{1}r_{1}=\rho_{1}/\beta_{1}^{2};r_{1}^{T}W_{2}r_{1}=\rho_{2}/\beta_{2}^{2};r_{2}^{T}W_{1}r_{2}=\rho_{1}/k\beta_{1}^{2};r_{2}^{T}W_{2}r_{2}=\rho_{2}/k\beta_{2}^{2};\\ r_{1}^{T}W_{1}r_{2}=0;r_{1}^{T}W_{2}r_{2}=0;r_{1}^{T}W_{1}t_{L1}=0;r_{1}^{T}W_{2}t_{L2}=0;\\ r_{2}^{T}W_{1}t_{L1}=0;r_{2}^{T}W_{2}t_{L2}=0;t_{L1}^{T}W_{1}t_{L1}=\rho_{1};t_{L2}^{T}W_{2}t_{L2}=\rho_{2} \end{array}$$

Establishing simultaneous equations with the first six equations in Equation (5) and utilizing the orthogonality of $r_{1}$ and $r_{2}$, we have:

(6) $$\begin{array}{l} r_{1}^{T}W_{1}r_{1}=kr_{2}^{T}W_{1}r_{2};r_{1}^{T}W_{2}r_{1}=kr_{2}^{T}W_{2}r_{2};r_{1}^{T}W_{1}r_{2}=0;r_{1}^{T}W_{2}r_{2}=0;\\ r_{2}^{T}r_{2}=1;r_{1}^{T}r_{1}=1;r_{1}^{T}r_{2}=0 \end{array}$$

Non-linear optimization is adopted to solve Equation (6). Thereafter, $r_{1}$ and $r_{2}$ can be solved directly. According to $R_{L1}=R_{L2}=[\begin{array}{ccc} r_{1} & r_{2} & r_{1}\times r_{2} \end{array}]$, we obtain $R_{L1}$ and $R_{L2}$. Similarly, the solution of $R_{R1}=R_{R2}$ can be determined.

Taking the target as a medium, the transformation matrix can be obtained as follows:

(7) $$\left[\begin{array}{cc} R_{\mathrm{LR}} & t_{\mathrm{LR}}\\ 0 & 1 \end{array}\right]=\left[\begin{array}{cc} R_{R1} & t_{R1}\\ 0 & 1 \end{array}\right]{\left[\begin{array}{cc} R_{L1} & t_{L1}\\ 0 & 1 \end{array}\right]}^{-1}$$

According to Equation (7), we have the final expression of $R_{\mathrm{LR}}$, which is shown in Equation (8) as follows:

(8) $$R_{\mathrm{LR}}=R_{R1}R_{L1}^{-1}$$

#### 2.2.2. Solving $t_{\mathrm{LR}}$

Establishing simultaneous equations with the last four equations in Equation (5) yields the following expression:

(9) $$\left\{ \begin{array}{l} r_{1}^{T}W_{1}t_{L1}=0\\ r_{2}^{T}W_{1}t_{L1}=0\\ r_{1}^{T}W_{2}t_{L2}=0\\ r_{2}^{T}W_{2}t_{L2}=0 \end{array}\right.$$

Given that Equation (9) has a typical form of AX = 0, we cannot obtain a unique non-zero solution $t_{L1}$ and $t_{L2}$ by solving Equation (9) directly. Upon analyzing Equation (9), $t_{L1}$ and $t_{L2}$ are the center of $e_{L1}$ and $e_{L2}$, respectively, which are the coordinates of the origin point of $O_{1}x_{1}y_{1}z_{1}$ and $O_{2}x_{2}y_{2}z_{2}$, respectively. Suppose that ${\tilde{t}}_{L1}$ and ${\tilde{t}}_{L2}$ are the unit vectors from the origin point of $O_{c1}x_{c1}y_{c1}z_{c1}$ to the origin point of $O_{1}x_{1}y_{1}z_{1}$ and $O_{2}x_{2}y_{2}z_{2}$, we have:

(10) $$\left\{ \begin{array}{l} {\tilde{t}}_{L1}=(W_{L1}^{T}r_{1}\times W_{L1}^{T}r_{2})/\left\|\left(W_{L1}^{T}r_{1}\times W_{L1}^{T}r_{2}\right)\right\|\\ {\tilde{t}}_{L2}=(W_{L2}^{T}r_{1}\times W_{L2}^{T}r_{2})/\left\|\left(W_{L2}^{T}r_{1}\times W_{L2}^{T}r_{2}\right)\right\| \end{array}\right.$$

Similarly, the rotation matrix $R_{R1}=R_{R2}$ and the translation vectors ${\tilde{t}}_{R1},{\tilde{t}}_{R2}$ can be solved according to the abovementioned method.

**Figure 3:**
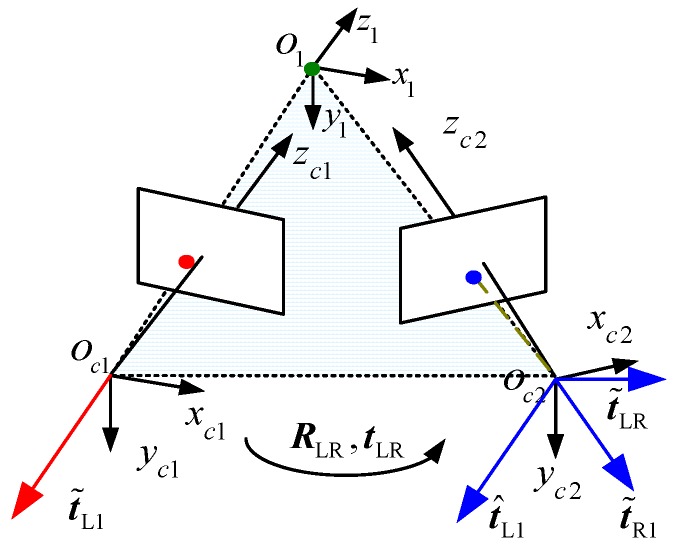
Process to solve ${\tilde{t}}_{\mathrm{LR}}$.

Let ${\tilde{t}}_{\mathrm{LR}}$ denote the unit vector from the origin point of $O_{c1}x_{c1}y_{c1}z_{c1}$ to the origin point of $O_{c2}x_{c2}y_{c2}z_{c2}$. As shown in Figure 3, ${\tilde{t}}_{L1}$, ${\tilde{t}}_{R1}$ and ${\tilde{t}}_{\mathrm{LR}}$ lie on a plane.

According to the coplanarity constraint, we have:

(11) $${\left({\hat{t}}_{R1}\times {\tilde{t}}_{L1}\right)}^{T}\cdot {\tilde{t}}_{\mathrm{LR}}=0$$

where ${\hat{t}}_{L1}=R_{\mathrm{LR}}{\tilde{t}}_{L1}$. Suppose that $v={\left({\hat{t}}_{R1}\times {\tilde{t}}_{L1}\right)}^{T}$, the coplanarity constraint can be rewritten as a homogeneous equation in ${\tilde{t}}_{\mathrm{LR}}$:

(12) $$v\cdot {\tilde{t}}_{\mathrm{LR}}=0$$

If *n* sets of images of the target are observed, by stacking *n* such equations as Equation (12), we have:

(13) $$V\cdot {\tilde{t}}_{\mathrm{LR}}=0$$

where V is an *n* × 3 matrix. If *n* ≥ 3, a unique solution ${\tilde{t}}_{\mathrm{LR}}$ can be obtained up to a scale factor. Unitizing the solution, we have the unit vector ${\tilde{t}}_{\mathrm{LR}}$. Given that $t_{\mathrm{LR}}=k_{\mathrm{LR}}{\tilde{t}}_{\mathrm{LR}}$, Equation (1) can be rewritten as follows:

(14) $$\left\{ \begin{array}{l} {\tilde{x}}_{L}={\tilde{z}}_{L}u_{L}/f_{L}\\ {\tilde{y}}_{L}={\tilde{z}}_{L}v_{L}/f_{L}\\ {\tilde{z}}_{L}=(f_{L}(f_{R}{\tilde{t}}_{x}-X_{R}{\tilde{t}}_{z}))/(u_{R}(r_{7}u_{L}+r_{8}v_{L}+f_{L}r_{9})-f_{R}(r_{1}u_{L}+r_{2}v_{L}+f_{L}r_{3})) \end{array}\right.$$

where ${\tilde{t}}_{\mathrm{LR}}={\left[{\tilde{t}}_{x},{\tilde{t}}_{y},{\tilde{t}}_{z}\right]}^{T}$.

According to Equation (14), we can obtain the coordinate of a feature point in 3D reconstruction up to a scale factor k, that is, $\left[x_{L},y_{L},z_{L}]=k[{\tilde{x}}_{L},{\tilde{y}}_{L},{\tilde{z}}_{L}\right]$. In detail, $\left[x_{L},y_{L},z_{L}\right]$ is the actual coordinates of feature point, where $\left[{\tilde{x}}_{L},{\tilde{y}}_{L},{\tilde{z}}_{L}\right]$ is the normalized coordinates of feature point up to the scale factor k. To solve k, we reconstruct the 3D coordinates of all the feature points that lie on the ellipse in $O_{c1}x_{c1}y_{c1}z_{c1}$. Using the plane fitting method, the coefficients of the two plane equations of the target can be determined as follows:

(15) $$\left\{ \begin{array}{l} a_{1}{\tilde{x}}_{L1}+b_{1}{\tilde{y}}_{L1}+c_{1}{\tilde{z}}_{L1}+{\tilde{d}}_{1}=0\\ a_{2}{\tilde{x}}_{L2}+b_{2}{\tilde{y}}_{L2}+c_{2}{\tilde{z}}_{L2}+{\tilde{d}}_{2}=0 \end{array}\right.$$

where $\left[a_{1},b_{1},c_{1},{\tilde{d}}_{1}\right]$ and $\left[a_{2},b_{2},c_{2},{\tilde{d}}_{2}\right]$ denote the coefficients of the two plane equations of the target when the scale factor k is unknown.

Similarly, the plane equations can be determined by fitting the coordinates of all the characteristic points in 3D reconstruction as follows:

(16) $$\left\{ \begin{array}{l} a_{1}x_{L1}+b_{1}y_{L1}+c_{1}z_{L1}+d_{1}=0\\ a_{2}x_{L2}+b_{2}y_{L2}+c_{2}z_{L2}+d_{2}=0 \end{array}\right.$$

where $d_{1}=k{\tilde{d}}_{1}$, $d_{2}=k{\tilde{d}}_{2}$.

Given that the two planes of target are parallel to each other, the actual distance *D* between two planes can be solved as the absolute of the difference between the distance between the origin of the left camera and two planes. According to Equation (15), the distance between the origin of the left camera and the plane can be solved up to the scale factor k. Thus, we have the normalized distance $\tilde{D}$ as follows:

(17) $$\tilde{D}=\left|\frac{\left|{\tilde{d}}_{1}\right|}{\left\|a_{1}^{2}+b_{1}^{2}+c_{1}^{2}\right\|}-\frac{\left|{\tilde{d}}_{2}\right|}{\left\|a_{2}^{2}+b_{2}^{2}+c_{2}^{2}\right\|}\right|$$

Considering that actual distance D of two planes is known, the scale factor k is inferred as:

(18) $$k=D/\tilde{D}$$

In this case, the final scale factor k is the average of the entire scale factor. Thus, k is presented as follows:

(19) $$t_{\mathrm{LR}}=k{\tilde{t}}_{\mathrm{LR}}$$

#### 2.2.3. Non-Linear Optimization

Calibration error exists due to random noise and other disturbances. Hence, non-linear optimization is utilized to obtain the optimal solution of calibration results. We randomly sample several feature points from one stripe, and the matching points will be the intersection of the other stripe and corresponding epipolar line.

To improve the calibration accuracy, the target is placed at different positions. For each position, assume that $O_{1i}x_{1i}y_{1i}z_{1i}$ and $O_{2i}x_{2i}y_{2i}z_{2i}$ are the target coordinate systems under two parallel planes. For the feature points located on different target planes, we reconstruct their 3D coordinates under the corresponding target coordinate system. Then, the ellipse fitting method is adopted to obtain $Q_{1i}$ and $Q_{2i}$. From $Q_{1i}$ and $Q_{2i}$, we can solve the major axes $\alpha_{1i}$ and $\alpha_{2i}$ and minor axes $\beta_{1i}$ and $\beta_{2i}$, as well as the angles $\theta_{1i}$ and $\theta_{2i}$. According to the properties of elliptical stripes, the objective function is established as follows:

(20) $$e_{1}(a)=\min\sum_{i}^{n}\left(\left|\frac{\alpha_{1i}}{\beta_{1i}}-\frac{\alpha_{2i}}{\beta_{2i}}\right|+\left|\theta_{1i}-\theta_{2i}\right|\right)$$

where $a=(R_{\mathrm{LR}},t_{\mathrm{LR}},R_{\mathrm{Li}}^{1},t_{\mathrm{Li}}^{1},t_{\mathrm{Li}}^{2})$ and $R_{\mathrm{Li}}^{1},t_{\mathrm{Li}}^{1}$ are the rotation matrix and transformation vector, respectively, from the left camera coordinate system to $O_{1i}x_{1i}y_{1i}z_{1i}$ at each position. $t_{\mathrm{Li}}^{2}$ is the transformation vector from the left camera coordinate system to $O_{2i}x_{2i}y_{2i}z_{2i}$, and *n* denotes the number of positions.

In each position, we reconstruct the 3D coordinates of the feature points under the coordinate system of BSVS. Then, the planar fitting method is utilized to obtain the equation of the left plane $\Pi_{Li}$ and right plane $\Pi_{Ri}$. Therefore, we obtain the second objective function based on the measurement distance and actual distance:

(21) $$e_{2}(a)=\min(\sum_{i}^{n}Dist(\Pi_{Li},\Pi_{Ri})-D)$$

where $Dist(\Pi_{1},\Pi_{2})$ is the distance of two planes under the coordinate system of BSVS, and *D* is the actual distance of the two parallel target planes.

According to the coplanarity constraint introduced in Section 2.2.2, we have the following objective function:

(22) $$e_{3}(a)=\min\sum_{i}^{n}\left({\left(t_{Ri}^{1}\times t_{Li}^{1}\right)}^{T}\cdot t_{\mathrm{LR}}+(t_{Ri}^{2}\times t_{Li}^{2}{)}^{T}\cdot t_{\mathrm{LR}}\right)$$

where *m* and *l* are the feature points in the two target planes, and *E* is the essential matrix of BSVS.

Thereafter, the final objective function is established as follows:

(23) $$e(a)=e_{1}(a)+e_{2}(a)+e_{3}(a)$$

Thus, the optimal solution of $R_{\mathrm{LR}}$ and $t_{\mathrm{LR}}$ under the maximum likelihood criteria can be solved via non-linear optimization methods (e.g., Levenberg–Marquardt algorithm [23]).

## 3. Discussion

**Figure 4:**
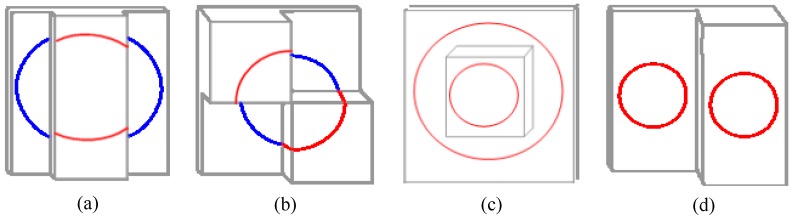
Combination forms of laser and target. (**a**) Single ring stripe laser and parallel planar target; (**b**) Single ring stripe laser and parallel planar target; (**c**) Concentric double ring stripe laser and parallel planar target; (**d**) Multiple ring stripe laser and parallel planar target.

The two geometric properties of projected elliptical stripe introduced in Section 2.2 comprise the core idea of the proposed method. Notably, various methods are available to obtain the elliptical stripes, such as the use of different forms of lasers or projector to project elliptical stripes on a target plane. Hence, equations in the form of Equation (5) are available to solve the rotation matrix and transformation vector of BSVS. The calibration form used in this study is the simplest form of the proposed method. If the axes of the projected light cone in each case remain parallel to each other, the elliptical stripes embody the geometric properties whether the divergence angle of the projective tool is a constant or not. Figure 4 shows several calibration forms for the proposed method.

The lasers shown in Figure 4 are easy to purchase, and the lasers with suitable wavelength and pattern according to the actual condition can be chosen. The BSVS is usually equipped with optical filter, so capturing an ordinary target clearly is difficult. The proposed method adopts the images captured by the strong laser. Thus, this method works much better under complex light conditions such as strong light, dim light, and non-uniform light. In comparison with common methods, the proposed method is more suitable for outdoor online calibration.

## 4. Experiment

### 4.1. Simulation Experiment

Simulation is performed to validate the efficiency of the proposed method. Image noise, distance of the two target planes, and size of the projected elliptical stripe considerably affect calibration accuracy when the BSVS is calibrated using the proposed method. Hence, simulation is performed based on the above factors. The conditions of the simulation experiments are as follows: camera resolution of 1628 pixels × 1236 pixels, focal length of 16 mm, field of view is 400 mm × 300 mm, placement position is approximately 600 mm away from the BSVS, $r_{\mathrm{LR}}$ is [0.0084, 0.6822, 0.0416], and $t_{\mathrm{LR}}$ is [−449.6990, −5.6238, 180.8245]^T^. Calibration accuracy is evaluated using the root mean square errors (RMSEs) of $r_{x}$, $r_{y}$, $r_{z}$, $t_{x}$, $t_{y}$ and $t_{z}$, as well as the deviation between the 3D reconstruction and actual coordinates of the feature points.

#### 4.1.1. Impact of Image Noise on Calibration Accuracy

**Figure 5:**
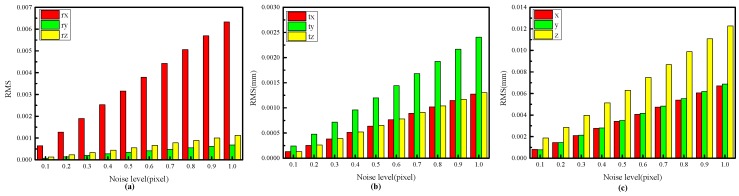
RMSEs of the extrinsic parameters based on the proposed method. (**a**) RMSEs of *r_x_*, *r_y_* and *r_z_* at different noise levels; (**b**) RMSEs of *t_x_*, *t_y_* and *t_z_* at different noise levels; (**c**) RMSEs of the 3D coordinates of the feature points at different noise levels.

In the experiment, the distance between the two target planes is 60 mm. The target is placed at 15 different positions in each experiment, and a total of 100 independent experiments are performed at each noise level. Gaussian noise with zero mean and standard deviation of 0.1–1 pixel with an interval of 0.1 pixel is added to the feature points. As shown in Figure 5, the calibration accuracy decreases linearly with increasing image noise. In general, the calibration accuracy is high even with a relatively high noise level.

#### 4.1.2. Impact of Distance between Two Target Planes on Calibration Accuracy

**Figure 6:**
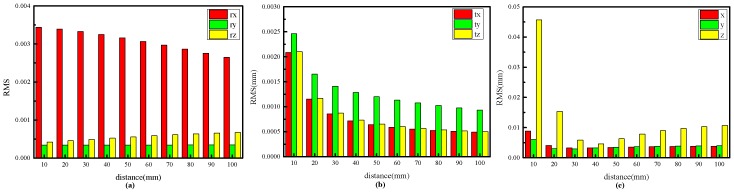
RMSEs of the extrinsic parameters based on the proposed method. (**a**) RMSEs of *r_x_*, *r_y_* and *r_z_* at different distance levels; (**b**) RMSEs of *t_x_*, *t_y_* and *t_z_* at different distance levels; (**c**) RMSEs of the 3D coordinates of the feature points at different distance levels.

In the experiment, the distance of two target planes is 60 mm. The target is placed at 15 different positions in each experiment, and a total of 100 independent experiments are performed at each distance level. Gaussian noise with zero mean and standard deviation of 0.5 pixel is added to the feature points. The distance between the two target planes ranges from 10 mm to 100 mm with an interval of 10 mm. As shown in Figure 6a,b, the RMSEs of $r_{x}$, $t_{x}$, $t_{y}$ and $t_{z}$ decrease as the distance levels increase, whereas the RMSEs of $r_{y}$ and $r_{z}$ increase as the distance levels increase. As shown in Figure 6c, the calibration accuracy increases remarkably with rising distance level in the range of 10–40 mm but gradually decreases when distance level increases in the range of 40–100 mm. Based on the above analysis, the improvement in calibration accuracy is not entirely related to the increase in distance level. High accuracy can be obtained when the ratio of field of view to the distance between two target planes is 10 (400 mm/40 mm).

#### 4.1.3. Impact of Elliptical Stripe Size on Calibration Accuracy

**Figure 7:**
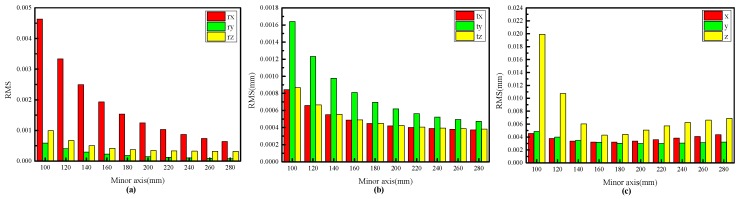
RMSEs of the extrinsic parameters based on the proposed method. (**a**) RMSEs of *r_x_*, *r_y_* and *r_z_* at different distance levels; (**b**) RMSEs of *t_x_*, *t_y_* and *t_z_* at different minor axis length levels; (**c**) RMSEs of the 3D coordinates of the characteristic points at different minor axis length levels.

In the experiment, the distance between two target planes is 60 mm. The target is placed at 15 different positions in each experiment, and a total of 100 independent experiments are performed at each size level. Gaussian noise with zero mean and standard deviation of 0.5 pixel is added to the feature points. The ratio of the major axes to the minor axes of the elliptical stripe in space is 1.1, and the length of minor axes is from 100 mm to 280 mm with an interval of 20 mm. As shown in Figure 7a,b, the RMSEs of extrinsic parameters decrease as the size levels increase. However, according to the reconstruction errors shown in Figure 7c, the calibration accuracy increases substantially with rising size level in the range of 100–160 mm but gradually decreases when the distance level increases in the range of 160–280 mm. For the proposed method, the most accurate calibration results do not necessarily contribute to the best calibration accuracy. From Figure 7c, the proposed method yields optimal calibration accuracy when the ratio of field of view to the distance between two target planes is approximately 2.5 (400 mm/160 mm).

### 4.2. Physical Experiment

Zhang’s method is widely used in camera calibration due to its convenience and efficiency. Hence, we compare the proposed method with Zhang’s method. In practice, Zhang’s method is flexible in application, and even a printed checkerboard paper is feasible in calibration. The calibration errors of Zhang’s method mainly come from two parts, namely, the manufacture error of the target and the location error of the image feature points [24]. For Zhang’s method, an important requirement of the checkerboard target is that the length of each grid must be equivalent and known. Thereafter, the calibration accuracy would decrease drastically when the target accuracy is not high. The normal checkerboard target and the light-emitting planar checkerboard target are the most commonly used targets for Zhang’s method; meanwhile, it is difficult to achieve high accuracy manufacturing for checkerboard. On the contrary, the double planar target can easily ensure high production quality with low cost, and the laser is easily obtained.

The calibration accuracy of Zhang’s method relies heavily on the extraction accuracy of the feature points of the target. When the lighting condition is unideal, the calibration image quality via Zhang’s method is poor with respect to the proposed method. Since the proposed method adopts strong laser stripes, it is easy to obtain the clear and stable calibration images. Steger method is used in the proposed method to extract laser stripe. Steger method is precise and stable when lighting changes, and it is used widely in complex situations and outdoor measurements. The following experiments are conducted to further prove the validity and stability of the proposed method, and show its’ superiority in application under complex circumstances.

#### 4.2.1. Performance of Different Targets in Complex Light Environments

In this section, the advantages and disadvantages of the proposed method and Zhang’s method are evaluated in complex lighting conditions, such as dim light and strong light. In the following experiments, a normal planar checkerboard target and a light-emitting planar checkerboard target are used in Zhang’s method, and a double parallel planar target is used in the proposed target.

**Figure 8:**
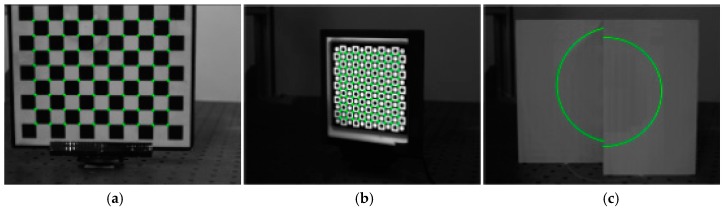
Calibration images based on two methods in the good light environment. (**a**) Calibration images of the normal checkerboard target; (**b**) Calibration images of the light-emitting checkerboard target; (**c**) Calibration images of the double parallel planar target.

Calibration images obtained in good light environments when the proposed method and Zhang’s method are used are shown in Figure 8. As shown in Figure 8, all the characteristic points and the light stripes on three targets can be extracted.

**Figure 9:**
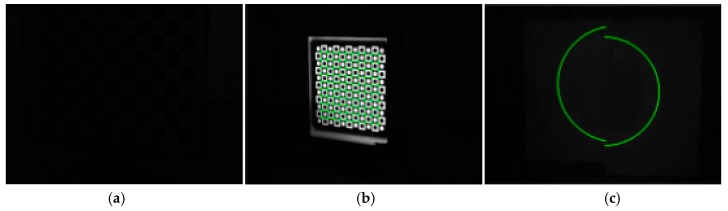
Calibration images based on two methods in the dim light environment. (**a**) Calibration images of the normal checkerboard target; (**b**) Calibration images of the light-emitting checkerboard target; (**c**) Calibration images of the double parallel planar target.

Calibration images obtained in dim light environment when the proposed method and Zhang’s method are used are shown in Figure 9. Generally, the methods used to obtain better calibration images are increasing the exposure time or aperture. Despite an increase of the exposure time or aperture, clear characteristic point images of the normal checkerboard target cannot be obtained in dim light environments. The light-emitting planar checkerboard target and double parallel planar target are feasible under dim lighting conditions. Consequently, the proposed method has certain advantages in the dim light environment. As shown in Figure 8, the characteristic points and the light stripes on the light-emitting planar checkerboard target and double parallel planar target can be extracted.

**Figure 10:**
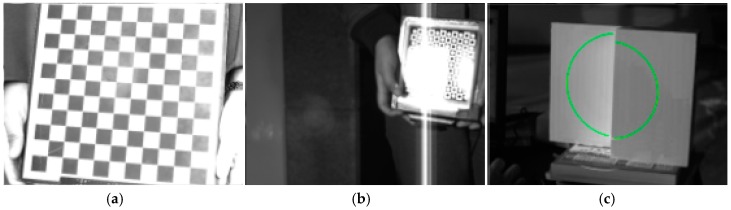
Calibration images based on two methods in the strong sunlight environment. (**a**) Calibration images of the normal checkerboard target; (**b**) Calibration images of the light-emitting checkerboard target; (**c**) Calibration images of the double parallel planar target.

Calibration images obtained in strong sunlight environment when the proposed method and Zhang’s method are used are shown in Figure 10. As shown in Figure 10, most characteristic points on the normal checkerboard target are difficult to obtain because of strong light. Strong light causes serious refraction on the surface of the light-emitting planar checkerboard target, and as a result, characteristic points on the refraction area cannot be extracted precisely. The proposed method adopts strong laser stripes to calibrate, and strong laser stripes are clear and stable in strong light environments. Obviously, the proposed method performs better than Zhang’s method.

According to the above experiments, the checkerboard targets are not feasible under the complex lighting conditions. Meanwhile, Zhang’s method performs poorly in strong light environments. On the contrary, the proposed method guarantees high accuracy and stability under complex lighting conditions.

#### 4.2.2. Extrinsic Calibration of BSVS

Two sets of physical experiments are performed, namely, the proposed method and Zhang’s method. Zhang’s method is widely used in camera calibration due to its convenience and efficiency. Hence, we compare the proposed method with Zhang’s method. 

**Figure 11:**
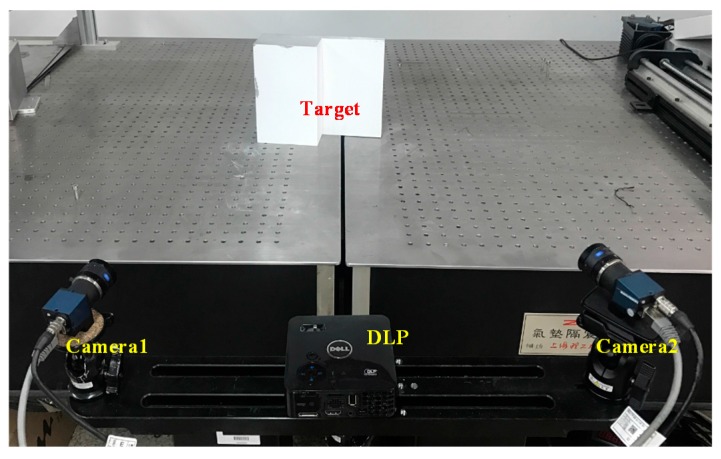
Stereo vision sensor and target.

As shown in Figure 11, two cameras are equipped with the same 16 mm optical lens. The resolution of the camera is 1628 pixels × 1236 pixels, the measurement distance is 600 mm, and the field of view is approximately 400 mm × 300 mm. The resolution of the projector (Dell, M110, Dell Computer Corporation, Round Rock, TX, USA) is 1280 pixels × 800 pixels.

MATLAB Toolbox in [25] is adopted to complete the intrinsic and extrinsic parameter calibrations of BSVS. A light-emitting planar checkerboard target is used in the physical experiments. The number of feature points on the target is 10 × 10, and the target accuracy is 5 µm. The intrinsic parameter calibration results of two cameras using Zhang’s method are shown in Table 1. 

The calibration process consists of the following steps: (1) the intrinsic and extrinsic parameters of BSVS are calibrated using Zhang’s method; (2) the calibration of the proposed method is implemented using the intrinsic parameters calibrated by Zhang’s method. The production accuracy of a double parallel planar target is 0.02 mm, and the distance between two target planes is 60.27 mm. The target is placed 15 times in each trial. 

**Figure 12:**
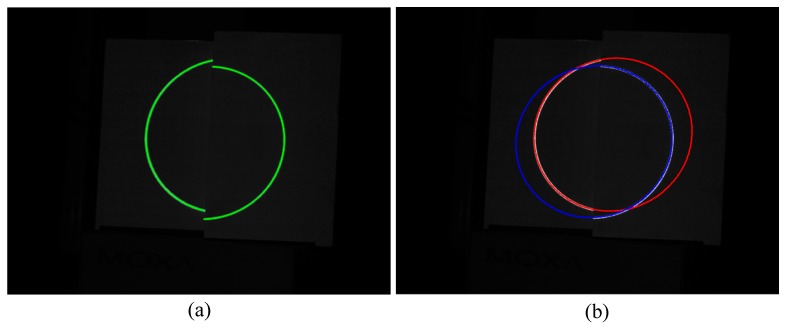
Result of processing the light stripes in the image. (**a**) Extraction of the center of the light stripes; (**b**) Ellipses obtained by ellipse fitting.

**Figure 13:**
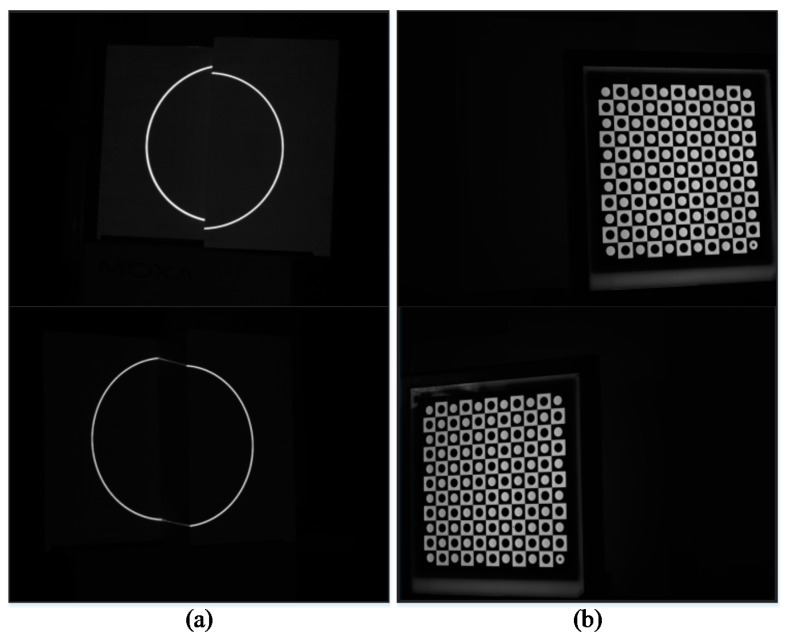
Images used in calibration via two methods. (**a**) Images used in calibration via the proposed method; (**b**) Images used in calibration via Zhang’s method.

The Steger method [26] is adopted to extract the center of the light stripes. Thereafter, the corresponding ellipse is obtained by the ellipse fitting method [27]. Figure 12 shows the results of processing the light stripes in the image. Images used in the two methods are shown in Figure 13.

Table 2 shows the comparison of the extrinsic parameters calibrated via the two methods. In general, the effects of the two extrinsic calibration methods show no significant difference.

#### 4.2.3. Evaluation of the Proposed Method

To further evaluate the proposed method, the light-emitting planar checkerboard target is placed five times before the BSVS. The feature points are the corner points of target, namely, the vertices of each grid on the target. The grid is a small square, and its length of side is 10 mm. The target accuracy is 1 µm, so the relative uncertainty of grid side length is ±0.01%. Obviously, the grid side length is fairly accurate. At each position, the 3D reconstruction coordinates of the feature points on target are computed based on the two methods. Table 3 shows the reconstruction results of five feature points at one of those positions.

**Figure 14:**
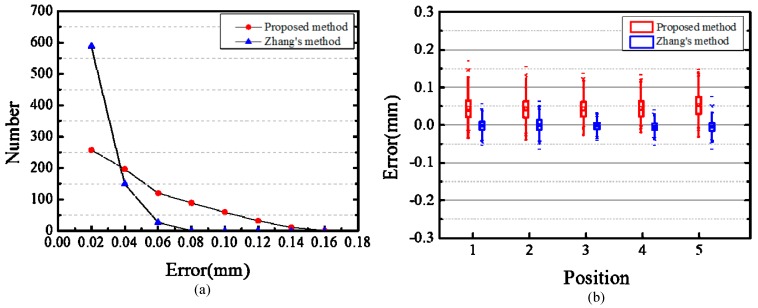
Reconstruction errors of light-emitting planar target via two methods. (**a**) Number of point pairs in different reconstruction error level via two methods; (**b**) Statistical distributions of the reconstruction error of the feature point pairs via two methods.

The measurement distance $d_{m}$ of the feature points is computed using the 3D reconstruction coordinates. The actual distance of the feature points on the target coordinate frame is denoted as $d_{t}$, which can be calculated with grid side length known. The deviation between measurement distance $d_{m}$ and actual distance $d_{t}$ is calculated as the reconstruction error Δd. Figure 14a shows the statistical diagram of the data in different reconstruction error levels, and Figure 14b illustrates the box chart showing the statistical analysis of reconstruction error.

From Figure 14a, most of the reconstruction errors based on Zhang’s method are relatively low. In the box chart, the two short horizontal lines above and below the error bar represent the maximum and minimum values of the data, respectively. As shown in Figure 14b, the deviation between the minimum reconstruction error and zero is relatively large when using the proposed method. The small rectangle in the error bar denotes the mean of the data. Compared with Zhang’s method, the mean reconstruction error using the proposed method considerably deviates from zero. The error bar shows the distribution of the data, and its lower and upper boundaries represent 25% and 75% of the data, respectively. Along the direction of the ordinate, the length of the error bar is relatively longer in the proposed method than in Zhang’s method. For Zhang’s method, the reconstruction error is more symmetric about zero, which means that the reconstruction errors are mainly close to zero. The reconstruction RMSEs of the proposed method and Zhang’s method are 0.03 mm and 0.02 mm, respectively. In terms of calibration accuracy, the proposed method is comparable with Zhang’s method

**Figure 15:**
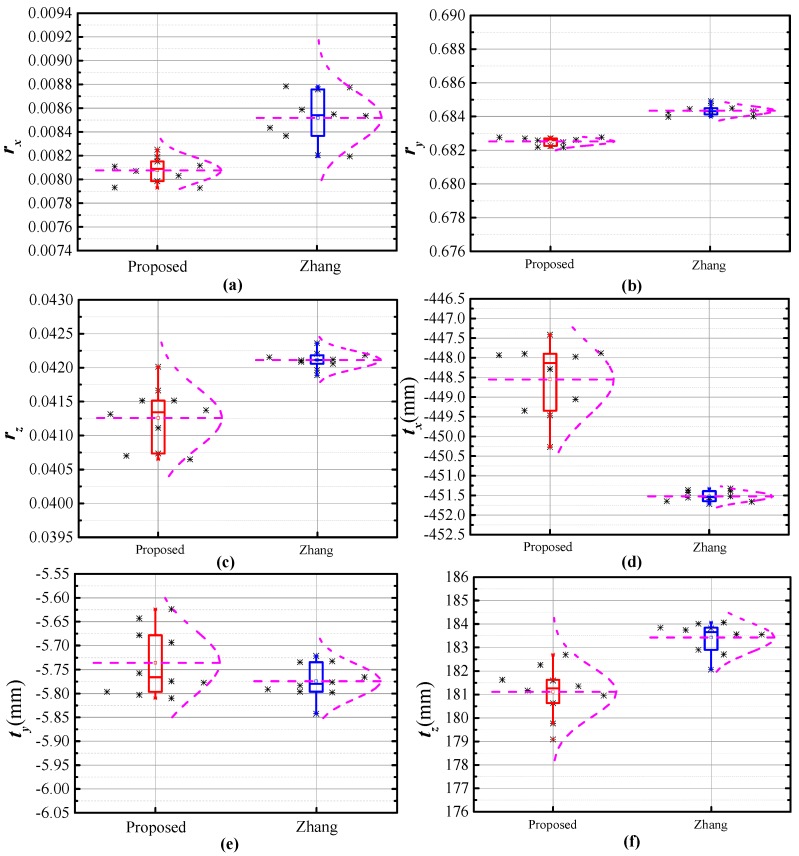
Repeatability of calibration results via two methods. (**a**) Repeatability of *r_x_* via two methods; (**b**) Repeatability of *r_y_* via two methods; (**c**) Repeatability of *r_z_* via two methods; (**d**) Repeatability of *t_x_* via two methods; (**e**) Repeatability of *t_y_* via two methods; (**f**) Repeatability of *t_z_* via two methods.

Stability is important for the evaluation of a calibration method. Hence, 10 sets of repetitive experiments are performed to validate the efficiency of the proposed method. For each method, 15 sets of images are randomly selected to calibrate the BSVS. Subsequently, repeatability analysis of the calibration parameters and calibration accuracy is conducted. Figure 15 shows the comparison of repeatability analysis of the calibration results. 

**Figure 16:**
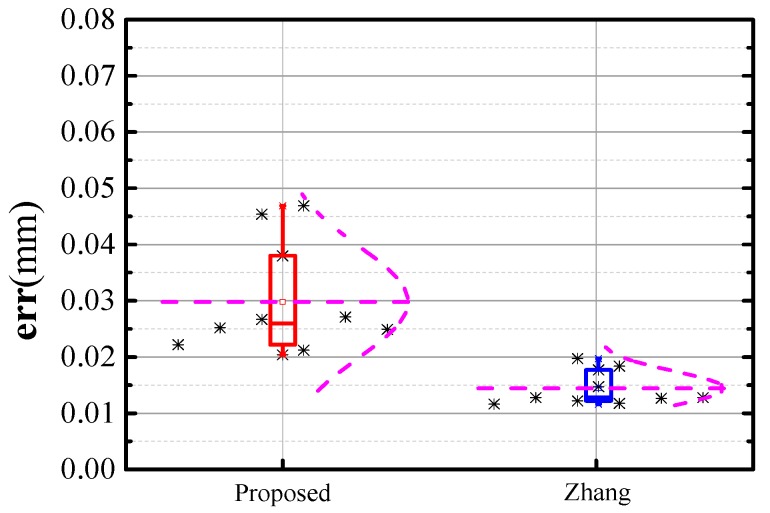
Repeatability of calibration accuracy error via two methods.

In Figure 15, the black asterisks represent the calibration parameters, the purple curves are the fitted normal distribution curves of the calibration parameters, and the thin horizontal lines in purple represent the mean calibration parameters. The shape of the normal distribution curve correlates with the standard deviation of the data. The curve is narrow and high when the standard deviation is low, whereas the curve with a relatively high standard deviation is flat and low. As shown in Figure 15b,f, the lengths of error bar of the proposed method is close to that of Zhang’s method, meanwhile, the fitted normal distribution curves are similar in shape. Hence, the stability of the proposed method is basically the same as that of Zhang’s method. It can be observed from Figure 15c–e that the dispersion of the calibration results of proposed method is high. However, the proposed method performs better in stability as shown in Figure 15a. Accuracy of the calibration method is determined by the entire extrinsic parameter. Hence, the efficiency of the calibration method cannot be evaluated well according to one parameter only. To further prove the stability of the proposed method, we calculated the RMS of the reconstruction errors to present the calibration accuracy of the two methods. Then, the contribution of calibration accuracy is analyzed as shown in Figure 16.

In Figure 16, the error bar represents the contribution of calibration accuracy via the two methods. The black asterisks are the entire calibration accuracy data. From the data, the calibration accuracy of Zhang’s method is approximately 0.02 mm, and that of the proposed method is close to 0.03 mm. In detail, the majority of calibration accuracy data of the proposed method is less than 0.03 mm. Along the direction of the ordinate, the length of the error bar of the proposed method is approximately twice that of Zhang’s method. Thus, the accuracy data of Zhang’s method is relatively concentrated. The thin horizontal lines in purple represent the mean calibration accuracy. By comparison, the mean calibration accuracy using Zhang’s method is close to 0.015 mm, which is approximately half that of the proposed method. In addition, the fitted normal distribution curve of Zhang’s method is relatively narrow and high, implying that the calibration accuracy of these methods is highly stable. Based on the above analysis, we make the following evaluation: Zhang’s method performs slightly better in stability and calibration accuracy, meanwhile, stability and calibration accuracy of both methods are relatively high.

The performance of the proposed method is slightly worse than Zhang’s method. However, some methods can be used in the calibration process to further improve calibration accuracy and stability. For instance, we can use multi-planar targets, project multiple elliptical stripes, and adopt enhanced non-linear optimization methods. The proposed method can adopt the feather point, which is not captured by the two cameras simultaneously. In general, the proposed method is slightly inferior to Zhang’s method but performs fairly well in practice. Moreover, the proposed method is convenient, flexible, and suitable for dynamic online calibration of BSVS. 

## 5. Conclusions

This paper presents an extrinsic calibration method based on unknown-sized elliptical stripe images. The proposed method avoids using high-accuracy target with certain-sized feature points. Strong light stripes are the core of the proposed method, which is suitable for calibration under complex circumstances. In addition, the proposed method performs well in calibration with an optical filter. The proposed method comes in various forms by flexibly combining the target and elliptical stripe, thereby guaranteeing relatively high calibration accuracy under different conditions. In practice, the planar target can easily ensure high production quality with low cost, and the laser is easily obtained. Several physical experiments validate the efficiency of the proposed method. In conclusion, the proposed method is valuable for practical extrinsic calibration of BSVS.

## Figures and Tables

**Table 1:** Intrinsic parameter calibration results of left and right cameras by Zhang’s method.

	*f_x_*	*f_y_*	*u*_0_ (pixel)	*v*_0_ (pixel)	*γ*	*k*_1_ (mm^−2^)	*k*_2_ (mm^−4^)
Left camera	3672.23	3672.87	833.11	631.99	8.46 × 10^−5^	−0.11	−0.05
Right camera	3673.59	3672.85	821.11	632.18	−1.59 × 10^−5^	−0.13	0.92

**Table 2:** Comparison of the extrinsic parameters.

	*r_x_*	*r_y_*	*r_z_*	*t_x_* (mm)	*t_y_* (mm)	*t_z_* (mm)
Proposed method	0.0084	0.6822	0.0416	−449.6990	−5.6238	180.8245
Zhang’s method	0.0082	0.6845	0.0421	−450.5520	−5.7329	183.8668

**Table 3:** Comparison of the 3D reconstruction results.

Index	Proposed Method			Zhang’s Method		
	*x* (mm)	*y* (mm)	*z* (mm)	*x* (mm)	*y* (mm)	*z* (mm)
1	100.430	−40.851	578.504	100.550	−40.899	579.197
2	100.732	−30.883	577.749	100.856	−30.921	578.464
3	101.028	−20.922	577.016	101.157	−20.949	577.753
4	91.072	−30.768	575.206	91.185	−30.806	575.923
5	91.676	−10.858	573.712	91.798	−10.872	574.472
